# A Low-Cost Instrumented Shoe System for Gait Phase Detection Based on Foot Plantar Pressure Data

**DOI:** 10.1109/JTEHM.2023.3319576

**Published:** 2023-09-26

**Authors:** Xinyao Hu, Qingsong Duan, Junpeng Tang, Gengshu Chen, Zhong Zhao, Zhenglong Sun, Chao Chen, Xingda Qu

**Affiliations:** Institute of Human Factors and Ergonomics, College of Mechatronics and Control EngineeringShenzhen University 47890 Shenzhen 518060 China; School of Science and EngineeringThe Chinese University of Hong Kong 26451 Shenzhen 518172 China; Department of OrthopedicsSchool of Traditional Chinese MedicineSouthern Medical University 70570 Guangzhou Guangdong 510515 China

**Keywords:** Instrumented shoe, gait phase detection, force-sensitive film, foot plantar pressure

## Abstract

This paper presents a novel low-cost and fully-portable instrumented shoe system for gait phase detection. The instrumented shoe consists of 174 independent sensing units constructed based on an off-the-shelf force-sensitive film known as the Velostat conductive copolymer. A zero potential method was implemented to address the crosstalk effect among the matrix-formed sensing arrays. A customized algorithm for gait event and phase detection was developed to estimate stance sub-phases including initial contact, flat foot, and push off. Experiments were carried out to evaluate the performance of the proposed instrumented shoe system in gait phase detection for both straight-line walking and turning walking. The results showed that the mean absolute time differences between the estimated phases by the proposed instrumented shoe system and the reference measurement ranged from 45 to 58 ms during straight-line walking and from 51 to 77 ms during turning walking, which were comparable to the state of art.Clinical and Translational Impact Statement—By allowing convenient gait monitoring in home healthcare settings, the proposed system enables extensive ADL data collection and facilitates developing effective treatment and rehabilitation strategies for patients with movement disorders.

## I. Introduction

Detection of gait phases within each gait cycle is fundamental for clinical gait analysis. Traditionally, the detection of gait phases is based on the ground reaction force obtained from the embedded force plate, or the lower body kinematics obtained from motion capture system [1]. However, these measurement systems are expensive and limited by space. Thus, traditional gait phase detection methods cannot be widely applied in clinical settings.

In recent years, many instrumented shoe systems have been developed to provide portable solutions for the gait phase detection. One popular solution is based on inertial measurement units (IMUs) since they are easy to be implemented, unobtrusive, and low-cost [2]. For example, Zhang et al. [3] proposed a real-time gait phase recognition method based on IMUs. Similarly, by using the lower limb kinematic data collected by two IMUs attached to the shank and foot, Sahoo et al. [4] developed a rule-based method for gait event detection during level-ground walking, stair ascent, and stair descent. Although various computational methods have been proposed [2], gait phase detection by IMUs remains challenging because the accuracy and reliability can be deteriorated by the sensor location and placement direction [5]. In addition, IMU signals can be affected by some gait-related factors, such as gait speed and gait progression direction (straight-line gait versus turning gait) [6].

In addition to IMUs, many instrumented shoe systems use isolated or discrete force sensors for gait phase detection [7]. For example, Godiyal et al., [8] presented a customized wearable force myography data acquisition system based on force resistive sensors to detect the events of heel strike and toe-off during overground and ramp walking. By using foot-force switches, Skelly and Chizeck [9] proposed a two-level gait phase detection algorithm and Agostini et al. [10] proposed a step-wise gait phase detection algorithm. Similarly, Bae [11] developed a hidden Markov model to detect gait phases based on four-foot force switches placed on the insole surface. Some force sensors, such as force sensing resistors (FSRs), are not durable when being exposed to long-lasting repeated forces, which could result in a short lifespan for gait phase detection applications [2]. More importantly, the accuracy and reliability of these gait phase detection methods is affected by the number and locations of the isolated sensors. There has been no consensus about the optimal number or locations of the force sensors for gait phase detection [12].

Foot plantar pressure information could be used in gait phase detection as it directly reflects foot-floor interactions [13]. In addition, the application of such information in gait phase detection may help avoid the effects caused by the number and locations of force sensors. Furthermore, from an instrument and measurement perspective, a system that can simultaneously measure foot plantar pressure data and detect gait phases can provide additional insights into the pathological deviation of gait [14]. Some low-cost foot plantar pressure sensing systems have been proposed recently. Crea et al. [15], for instance, developed a pair of foot pressure insoles equipped with 64 optoelectronic pressure sensors for gait phase detection. Later, Martini et al. [16] improved Crea et al.’s measurement system by using fewer pressure sensors to detect gait phases.

One limitation in the existing studies using plantar pressure information in gait phase detection is the limited number of pressure sensing units which was typically ranged from 4 to 64 [17]. With such a small number of sensing units, foot plantar pressure can only be estimated by computational models without high accuracy [18]. Increasing the number of pressure sensing units could be challenging because there could be the crosstalk effect among the matrix-formed sensing arrays.

There was also lack of robust gait phase detection algorithms based on the plantar pressure information. The existing gait phase detection algorithms can be generally categorized into two types, i.e., threshold-based and machine-learning-based [2]. In the threshold-based algorithms, a threshold that could be either static or dynamic should be pre-determined according to the domain knowledge [19], and a gait event of interest is considered to be detected once the observed variable exceeds the threshold. The advantages of the threshold-based algorithms were their simplicity and computational efficiency, which allowed them to be easily implemented in real-time applications [2]. However, these algorithms often had a comparably lower detection accuracy compared to the machine-learning-based algorithms. Various machine learning models including the support vector machine [4], artificial neural network [20], hidden Markov model [21], and convolutional neural network [22], have been used in gait phase detection applications. Compared to the threshold-based algorithms, the machine-learning-based algorithms can often achieve superior detection accuracy. However, these algorithms require a tremendous amount of data for model training and development, and they are generally more computational demanding than the threshold-based algorithms [2].

To address the above-mentioned limitations, this paper presents a novel low-cost, and fully portable instrumented shoe system for gait phase detection. The novelty of the present study lies in the following two aspects.

First, unlike most extant low-cost foot plantar measurement systems, the proposed instrumented shoe can offer a high resolution for pressure sensing, given that it consists of 174 independent sensing units. Such high sensing resolution not only provides more detailed information about the foot-floor interaction, but also improves the gait phase detection performance. It also enables gait phase detection applications no longer to be restricted by the numbers and locations of the discrete sensing units. The whole system costs approximately 30 US dollars. Such high cost-efficiency gives this system the potential to be used for remote and home settings.

Second, a customized gait phase detection algorithm was developed based on the foot plantar pressure data. This peak heuristic search algorithm was developed based on plantar pressure data with relevant domain knowledge of the foot-floor interaction at each critical gait event. Compared to the extant gait phase detection algorithms, the proposed method can detect the sub-gait phases with high accuracy and marginal time errors for both straight-line walking and turning walking. In addition, it has higher computational efficiency than machine learning methods.

The remainder of the paper is structured as follows. In Section II, the design of the instrumented shoe is described (Section II-A), followed by the description of the sensor characteristic tests (Section II-B). Then, the gait phase detection algorithms are introduced in Section II-C. The experiment carried out for system evaluation is described in Section II-D, and the evaluation process is detailed in Section II-E. The evaluation results are reported in Section III. The discussion is presented in Section IV. Finally, Section V summarizes this research.

## II. Methods

### A. Hardware Design

**FIGURE 1.**
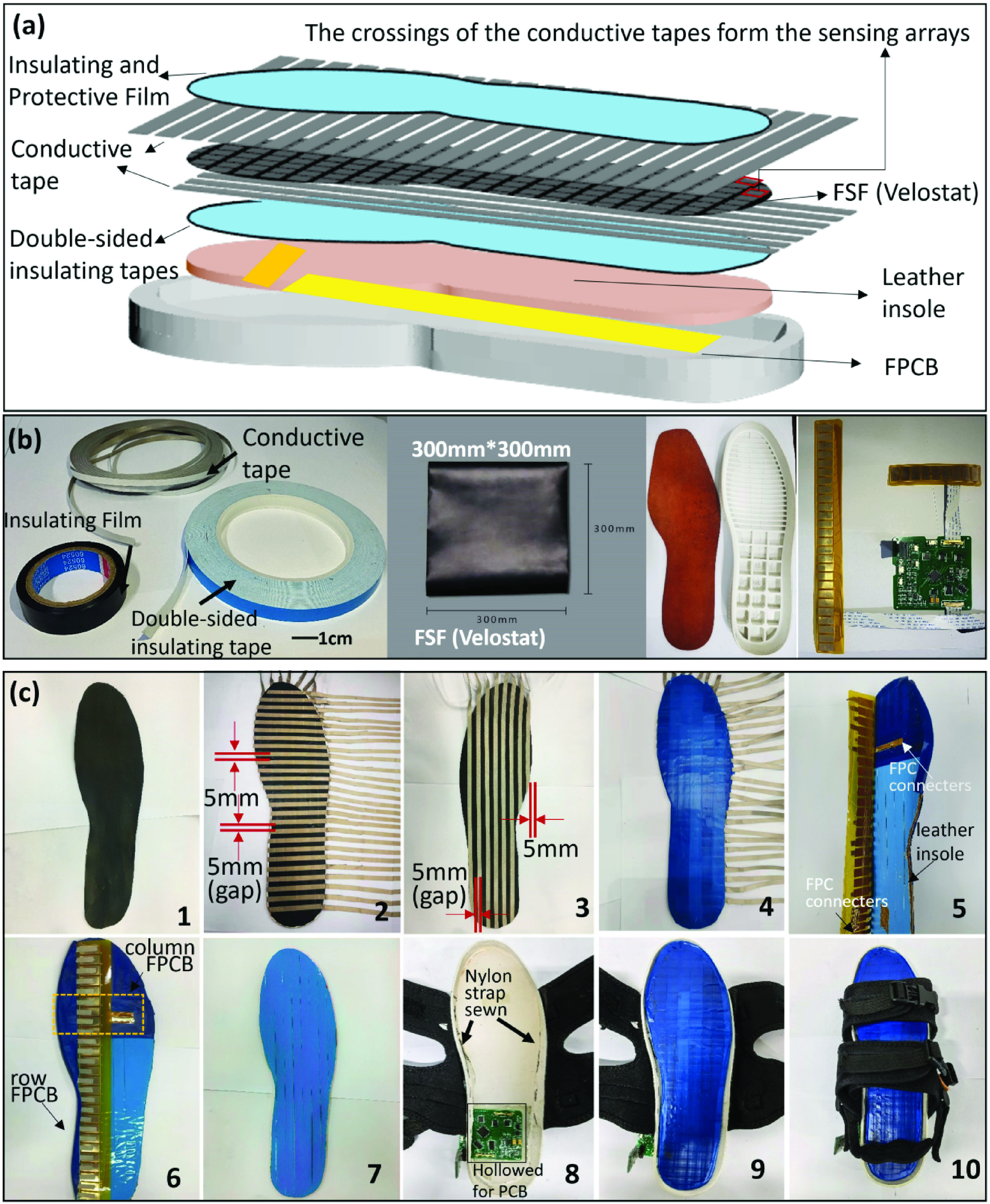
(a) Design concept of the pressure-sensitive insole; (b) the raw materials used for the insole; (c) the step-by-step illustration of the making procedure: 1 - the tailored FSF (velostat), 2 - the rows of the sensing array, 3- the columns of the sensing array (on the back side), 4 - the insulating and protective film cover, 5 and 6– the insole connected with FPCB, 7 - the appearance of the insole, 8 - the design of the sole, 9– the sole with the insole inserted, 10– the appearance of the instrumented shoe.

The design of the instrumented shoe is depicted in Fig. 1. The sensing arrays were constructed based on an off-the-shelf force-sensitive film (FSF) known as the Velostat conductive copolymer (Adafruit Industries Inc., US) [24]. The FSF was tailored according to the shape of a leather insole with the size of US 9 (length=265 mm, largest width=90mm) (Fig. 1(c)-1). One of the design goals was to provide high plantar pressure sensing resolution. Thus, a matrix structure of sensing arrays was created. Specifically, the FSF was sandwiched by two layers of conductive tapes (width=5mm) (Brand Co., Ltd., China). The upper layer of the conductive tapes (N=26) aligned parallelly in rows with a gap of 5mm in-between (Fig. 1(c)-1). Similarly, the lower layer of the conductive tapes (N=8) aligned parallelly in columns with the same gap width (Fig. 1(c)-1). This created a matrix of sensing arrays at the crossing points of the upper and lower conductive tapes. Some sensing arrays were cut off to meet the shape of the insole. Another advantage of such a matrix design with shared row and column lines is the computability with reduced numbers of inter-connection lines and I/O (input/output) pins. Overall, 174 sensing units were created with 32 I/O pins. This sandwiched film was then adhered onto the leather insole with double-sided insulating tapes made by Polymethylethacrylate (PPMA) and silicone adhesive (Brand Co., Ltd., China) (Fig. 1(c)-1).

The surface of the insole was covered with an insulating film made by flexible PVC (polyvinyl chloride) for protection. The end of the row conductive tapes and the end of the column conductive tapes were affixed onto the customized flexible printed circuit boards (FPCBs) (Fig. 1(c) -II-C-6) which were in turn connected to the control board (length=68.7 mm, width=56.6 mm) by flat flexible cable (FFC) and FFC-FPC connectors (Fig. 1(c)-1). The system was powered by a 3.7V-230mAh polymer Lithium-ion battery (size: 25mm $\times25$mm $\times4.5$mm) and could operate for at least three hours. An off-the-shelf soft rubber shoe sole was modified to accommodate the force sensing insole, the control board and battery (Fig. 1(c)-1). Nylon straps were sewn onto the shoe sole to endow the instrumented shoe with wearability (Fig. 1(c)-1). The total weight of this instrumented shoe was 392 g (Fig. 1(c)-1), similar to the weight of a normal sports shoe.

### B. The Design of the Control Board

**FIGURE 2.**
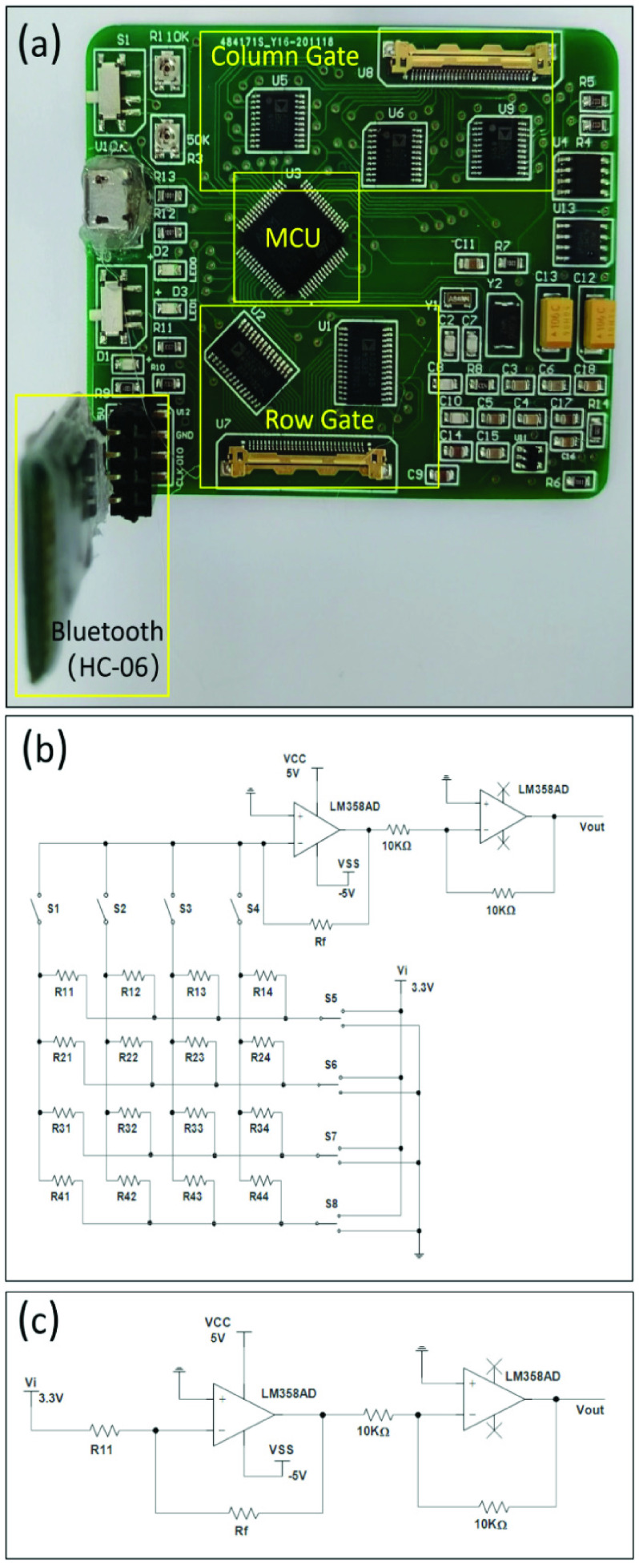
(a) The control board; (b) a zero potential circuit with 4-by-4 sensing arrays; (c) the equivalent circuit of the sensing array R11.

The design of the control board is depicted in Fig.2 (a). A micro control unit (MCU) (32-bit ARM Cortex, ARM Ltd., UK) was used for data processing and logging. A Bluetooth chip (HC-06, Wavesen Co. Ltd., China) was used for data transmission. Given the network structure of resistors in the pressure sensing arrays, the cross-talk effect inevitably exists, which means that the output of a certain sensing unit would be interfered by its nearby sensing units [25], [26]. To address the cross-talk effect, a zero potential method was implemented [27]. Each row of the sensing units was connected to a single-pole-double-throw (SPDT) switch (i.e., the row gate), and each column was connected to a single-pole- single-throw (SPST) switch (i.e., the column gate) (Fig. 2(b)). Such design allowed the selected row to be connected to a load resistor $\text{R}_{\mathrm {f}}$ (=10K ohm) and the selected column to be connected to the negative input of an operational amplifier (Opt-Amp), while the other columns and rows were connected to the ground.

Fig. 2(c) shows the equivalent circuit when the sensing unit R_11_ was selected to be assessed. By connecting the column switch S1 and the row switch S5 while keeping all the other switches off, R_11_ can be connected to the negative feedback path of the Op-Amp with all the other sensing units isolated from it. As a result, the resistance change of this sensing unit can be monitored directly by the output voltage ( $\text{V}_{\mathrm {out}}$) of the Opt-Amp (without the effect of other sensing units). Likewise, by manipulating the SPDT and SPST switches at the row and column gates, the resistance of any sensing unit $\text{R}_{\mathrm {ij}}$ can be isolated and assessed based on the measured Vout, Rf, and the input voltage $\text{V}_{\mathrm {i}}$ (which was set at 3.3 V) without the cross-talk effect by the following equation.

\begin{equation*}R_{ij} =\frac {V_{i}}{V_{out}}R_{f} \tag{1}\end{equation*}

Since $\text{R}_{\mathrm {ij}}$ and $\text{V}_{\mathrm {out}}$ had an inverse relationship, Vout was directly measured and converted into 12-bit digital data ranging from 0–4095 by the ADC module integrated in the MCU.

### C. Sensor Characteristics Tests

Two tests were carried out to characterize output response of the sensing units. Because the gait events detection algorithms were later developed based on the sensing output from the fore foot (SA1), foot arch (SA2) and heel (SA3), one sensing unit was randomly selected from each of these sub-plantar areas during the characteristic test. Thus, three sensing units in total were selected for testing.

**FIGURE 3.**
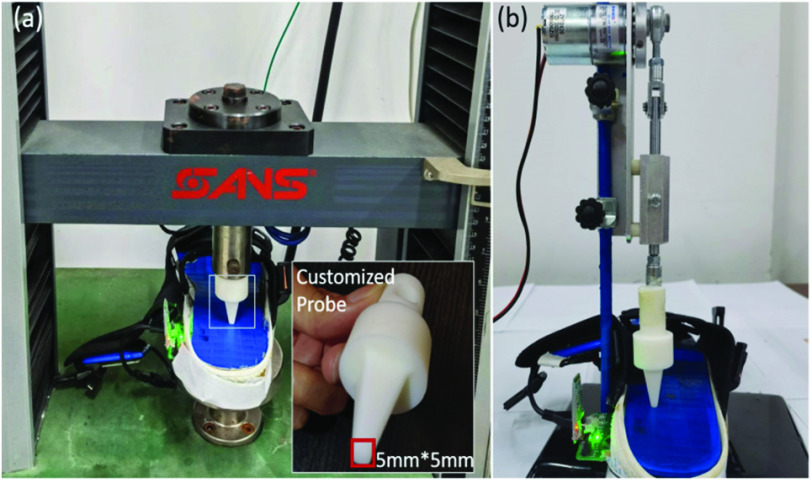
Experiment set-up of the sensor output characteristic test. (a) with constantly increased force; (b) with cyclically changed force.

The first test aimed to investigate how the sensor output changed under linearly increased force. Although such linearly changed force did not imitate the forces applied on the foot plantar area during gait, it can help quantify the relationship between the applied force and the sensor output. An omni-mechanical tester (SANS; MST System Co. Ltd., China) was used to apply linearly increased force on the insole. A customized testing probe was designed with the tipping area fitting the exact size of the sensing unit (length=width=5mm), as depicted in Fig. 3(a). Given the size of each sensing unit (25 mm $^{2}$), the foot plantar loading on this area is approximately between 5 and 9.5 N during normal walking and running [28]. Thus, the maximum loading level was set as 10N. The loading rate was set at 10N/s with the initial contacting force (i.e., the entry force) at 0.05N. The loading force was kept at 10N for five seconds.

The second test aimed to mimic the force applied on the foot plantar area during each gait cycle so as to investigate how the sensor output changed under cyclic force. The same testing probe was attached to a customized tester that was actuated by a motor with an eccentric rotor (Zheng Motor Co. Ltd., China). As depicted in Fig. 3(b), this tester can allow its shaft to generate a reciprocating motion with adjustable speed from 2 rpm to 200 rpm.

To mimic different levels of walking cadence, the motor speed was set at 40 rpm (i.e., 40 reciprocating motions per minute), 60 rpm, and 80 rpm, which simulated the cadence levels of 40, 60, and 80 strides per minute, respectively. The selected cadence levels were considered to be representative of slow walking speed, normal walking, and fast walking speed, respectively [29]. Similar to the aforementioned test, the motor was set to generate force approximately at 9.8 N to mimic the foot plantar loading on this area during gait [28]. Under each condition, the force was applied on the sensor unit for approximately 1.25 second per cycle (s/c), 1 s/c, and 0.75 s/c.

**FIGURE 4.**
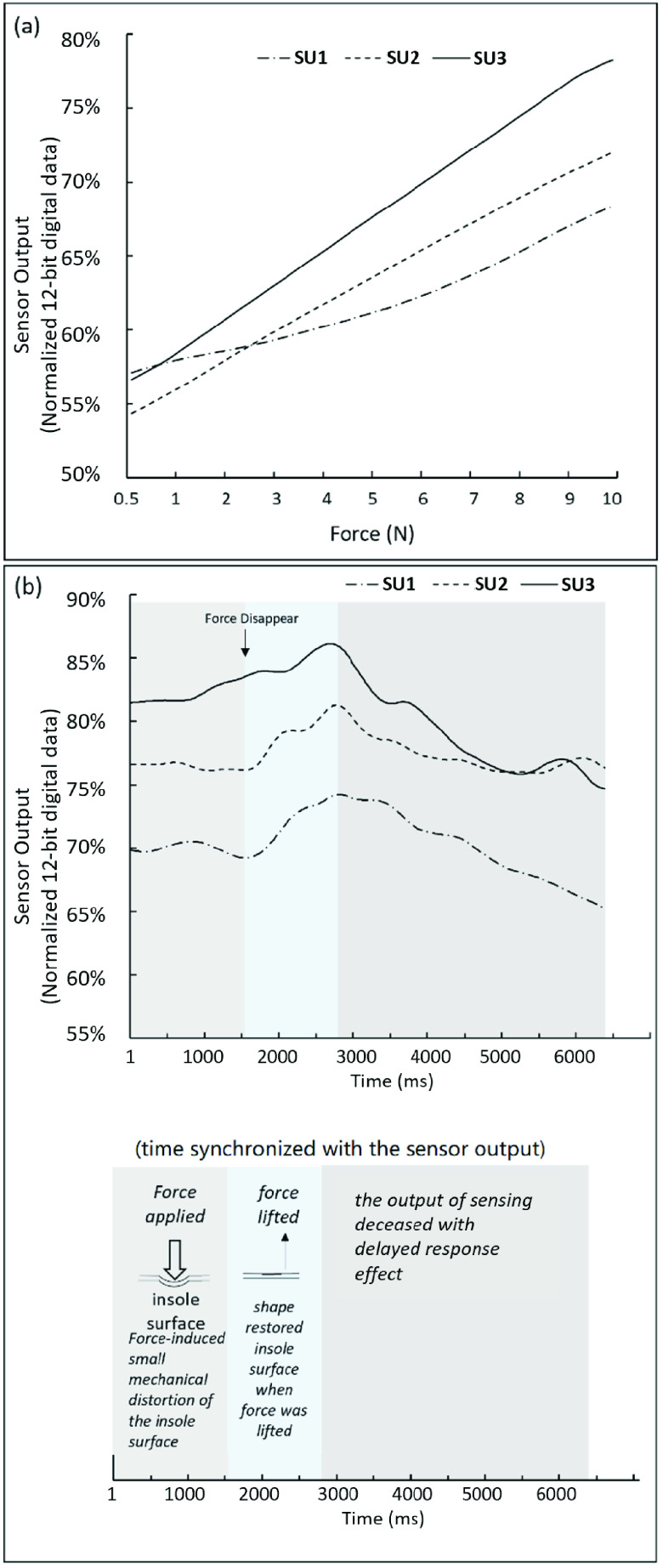
(a) An example of the sensor output response of three randomly selected sensing arrays, SU=sensing unit; (b) An example showing the delayed response effect when the force was no longer applied on sensing arrays. The sensor output was normalized from the 12-bit digital data ranging from 0-4095. Note: data shown in (a) and (b) were from different testing trials.

Furthermore, it was also notable that there existed a delayed response effect. As depicted in Fig. 4 (b), when the loading force was lifted from the surface of the insole, the sensor output did not decrease immediately. Instead, it kept increasing for approximately one second before starting to decrease. Such delayed response effect can possibly be attributable to the small mechanical distortion of the insole surface and the conductive tapes due to the applied normal force [30]. The shoe sole structure was elastic. The applied force can lead to a slight surface distortion, which made the conductive tapes bound with the FSF more tightly. When the force was removed and the affected surface area began to restore to its original shape, the sensor output increased at the beginning and then started to decrease. Thus, at this moment, this instrumented shoe system is not expected to be reliable for the measurement of the actual magnitude of the applied force.

**FIGURE 5.**
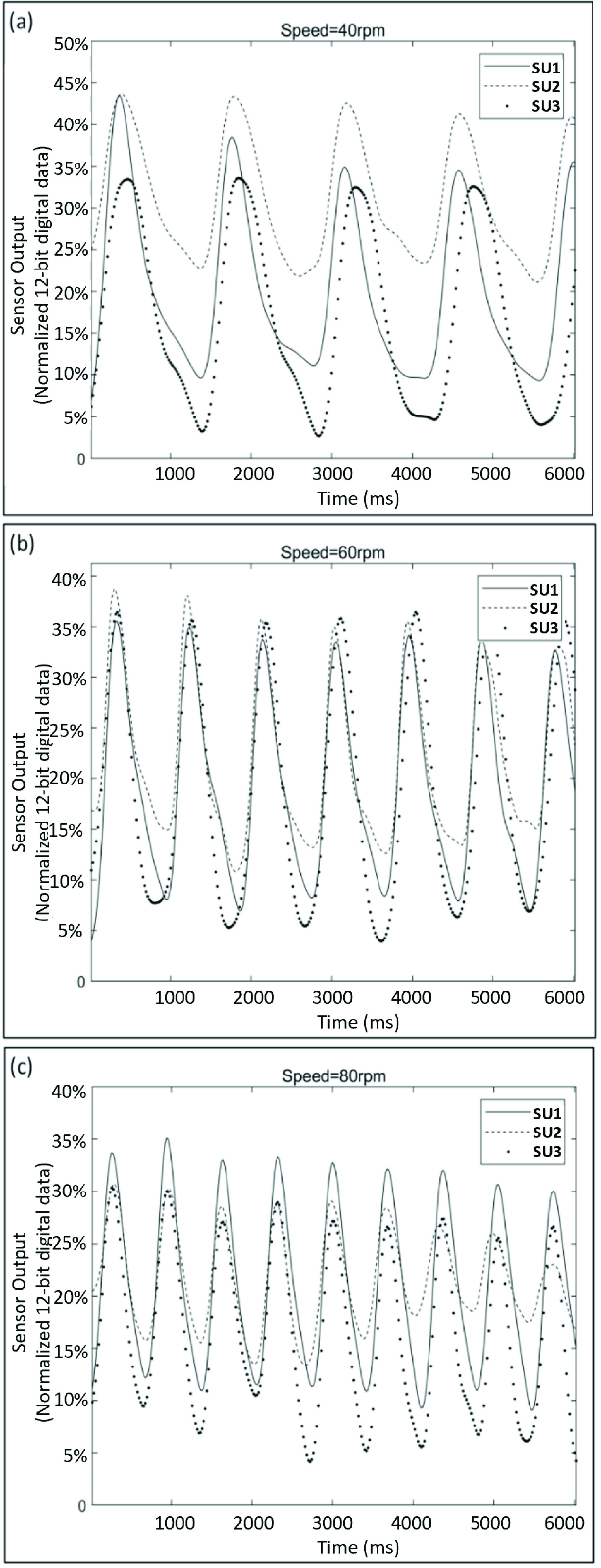
Sensor output response under cyclically changed force. (a) motor speed = 40 rpm; (b) motor speed = 60 rpm; (c) motor speed = 80 rpm; SU=sensing unit. rpm = reciprocating motion per minute. The sensor output was normalized from the 12-bit digital data ranging from 0-4095. The example sensor output response of the sensing unit (quantified by the voltage output) from the first test was shown in Fig. 4. The voltage output of each sensing unit increased almost linearly with respect to the applied force (Fig. 4(a)). Notably, the changing rate of the resistance for each sensing unit seemed diverse. This was likely due to the slightly uneven interface between FSF, the conductive tapes, and the leather insole.

The sensor output with respect to the cyclically applied forces were shown in Fig. 5, which demonstrated clearly cyclic pattens in response to the cyclic force applied with different frequencies. No apparent delayed response was observed in the sensor output, which suggested that the sensor output could be reliable when the force was applied on the sensing unit cyclically and briefly (from 0.75 second to 1.25 second). Nonetheless, it also showed that the output of each sensing unit seemed diverse, especially when the applied force lasted longer (i.e., 1.25 second). The variations in baseline resistance and sensor output are possibly caused by two main factors. One possible factor is the manufacturing variances. Slight differences in manufacturing processes can lead to variations in the conductive properties across different areas on the Velostat film. These variations can affect the baseline resistance of the sensing unit, leading to differences in the initial output. The second factor is the variations in the mechanical properties of each sensing unit, such as stiffness or stretchability, which are influenced by the conductive tapes on both sides of the Velostat film. Since the baseline resistance and output from a single sensing unit might not be consistent with the other sensing units, it is not reliable to detect gait events based on a single sensing unit alone.

#### 1) Gait Phase Detection Algorithm

**FIGURE 6.**
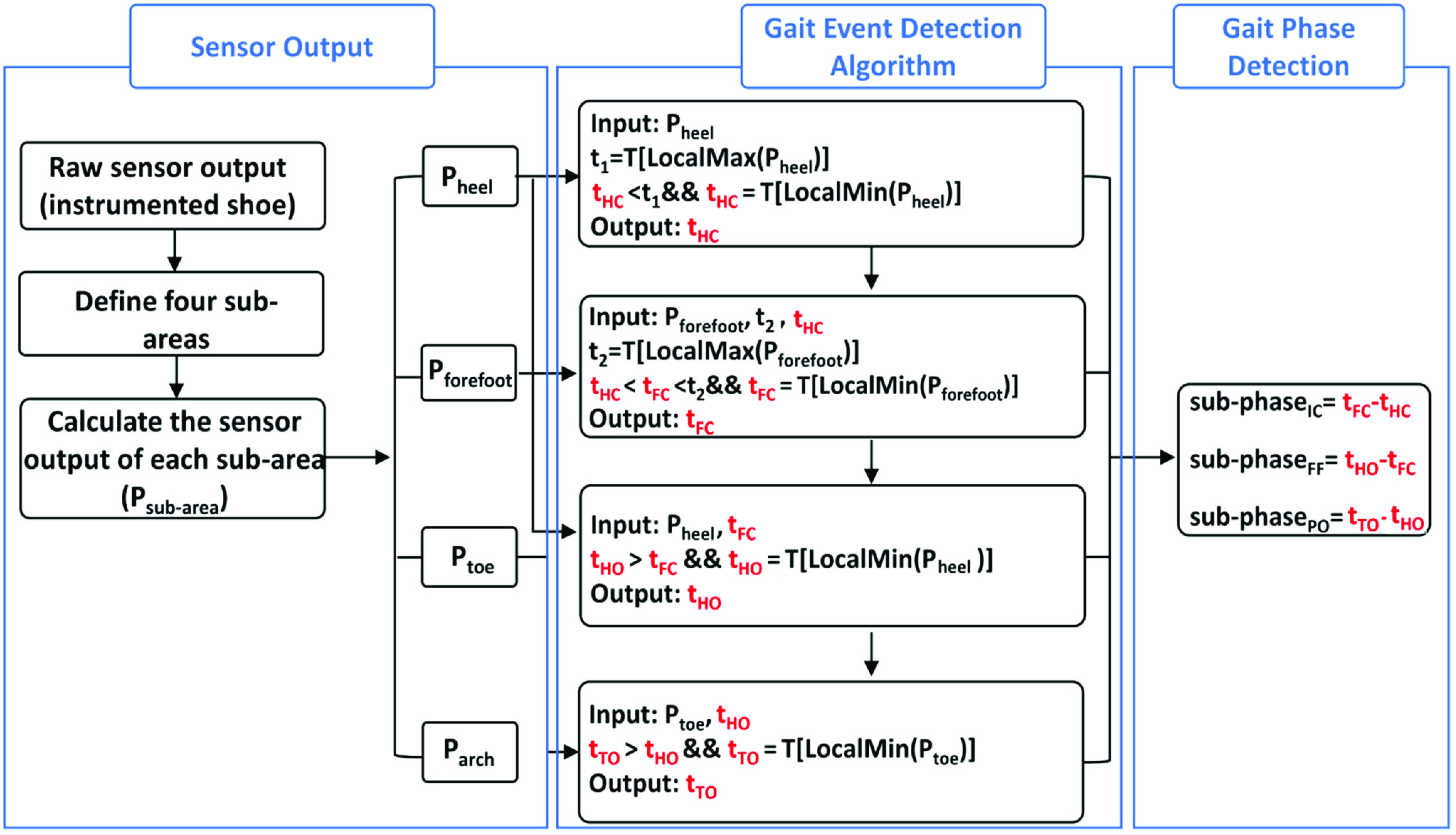
The block diagram illustrating the work flow of the proposed gait phase detection algorithm. Pheel, Pforefoot, Ptoe, Parch represent the calculated sensor outputs of the heel, forefoot and toe sub-areas, respectively; t1 is the moment of the first peak of the sensor output in the gait cycle from the heel sub-area; t2 is the moment of the first peak of the sensor output in the gait cycle from the forefoot sub-area; HC-heel contact; FC-Forefoot Contact; HO-Heel Off; TO-Toe Off.

A peak heuristic algorithm was developed based on the above-described characteristics of the sensing arrays. Since the resistance of the sensing arrays had different changing rates, instead of using the data from a single sensing unit, this algorithm was based on the average value of multiple sensing units from different foot plantar sub-areas. The use of the output from multiple sensing units allowed us to minimize the effect of resistance difference between the sensing units. The sensing units were divided into four main plantar sub-areas, including the heel, foot arch, fore foot, and toe based on the subdivision method introduced in an early study [31]. For each sub-area, the sensor output ( $P_{sub-area}$) was calculated by averaging the outputs of all the sensing units within this sub-area as follows,

\begin{equation*} {P}_{sub-area}=\frac {\sum \nolimits _{i=1}^{N} {P}_{i}}{N} \tag{2}\end{equation*}

where $P_{i}$ was the 12-bit digital output from each single sensing unit of the corresponding sub-area. As the relationship between $\text{P}_{\mathrm {i}}$ and the applied force is close to be linear (Fig. 4a), $\text{P}_{\mathrm {i}}$ can reflect the pressure under the corresponding sub-area. A gait cycle can be divided into two gait phases that are the stance phase and swing phase [1]. The stance phase can be further partitioned into three sub-phases, i.e., initial contact (IC), flat foot (FF), and push off (PO) [2]. These stance sub-phases are defined by key gait events including heel contact (HC), fore foot contact (FC), heel off (HO) and toe off (TO). Specifically, initial contact is defined by the interval between heel contact and fore foot contact, flat foot is defined by the interval between forefoot contact and heel off, and push off is defined by the interval between heel off and toe off. Thus, to determine durations of the stance subphases, key gait events must be detected. Fig. 6 illustrates the work flow of the proposed gait phase detection algorithm. The times of HC, FF, HO, and TO were determined by the sensor outputs ( $P_{sub-area}$) from different sub-areas. Specifically, to locate the time of HC within each gait cycle, the first peak in the gait cycle ( $t_{1}$) of the sensor output from the heel sub-area ( $P_{heel}$) was identified first,

\begin{equation*} t_{1}=T(\max _{local} P_{heel}) \tag{3}\end{equation*}

and the time of HC ( $_{tH}\text{C}$) was the moment of the local minimum right before the peak, as follows.

\begin{equation*}t_{\mathrm {HC}}=T\left ({\min _{local} P_{heel} }\right)(t_{\mathrm {HC}}< t_{1}) \tag{4}\end{equation*}

The obtained ${t} _{HC}$ was subsequently used to identify the time of FC within the gait cycle $(t_{FC})$, which was determined as the local minimum right before the first peak of the sensor output in the forefoot sub-area $(t_{2})$, as follows,

\begin{align*} t_{2}&=T(\max _{local}P_{forefoot}) \tag{5}\\ t_{\mathrm {FC}}&=T\left ({\min _{local} P_{forefoot} }\right)(t_{HC}< t_{\mathrm {FC}}< t_{2}) \tag{6}\end{align*}

Due to the delayed response effect, the sensor output of the heel sub-area could slightly increase at HO. Thus, the time of HO within a gait cycle ( $t_{HO}$) was identified at the time when the corresponding sensor output started to increase, i.e., the first local minimum in the sensor output of the heel sub-area after $t_{FC}$ as follows.

\begin{equation*}t_{\mathrm {HO}}=T\left ({\min _{local} P_{heel} }\right)(t_{\mathrm {HO}}>t_{FC}) \tag{7}\end{equation*}

Such delayed response effect was also observed at the time of TO. Thus, the time of TO ( $\text{t}_{\mathrm {TO}}$) was identified at the time of the first local minimum in the sensor output of the toe sub area after $\text{t}_{\mathrm {HO}}$, as follows.

\begin{equation*} t_{\mathrm {TO}}=T\left ({\min _{local} P_{toe} }\right)(t_{\mathrm {TO}}>t_{HO}) \tag{8}\end{equation*}

Subsequently, the durations of the sub-stance phases were calculated as follows.

\begin{align*} T_{\mathrm {IC}}&=t_{\mathrm {FC}}-t_{\mathrm {HC}} \tag{9}\\ T_{\mathrm {FF}}&=t_{\mathrm {HO}}-t_{\mathrm {FC}} \tag{10}\\ T_{\mathrm {PO}}&=t_{\mathrm {TO}}-t_{\mathrm {HO}} \tag{11}\end{align*}

**FIGURE 7.**
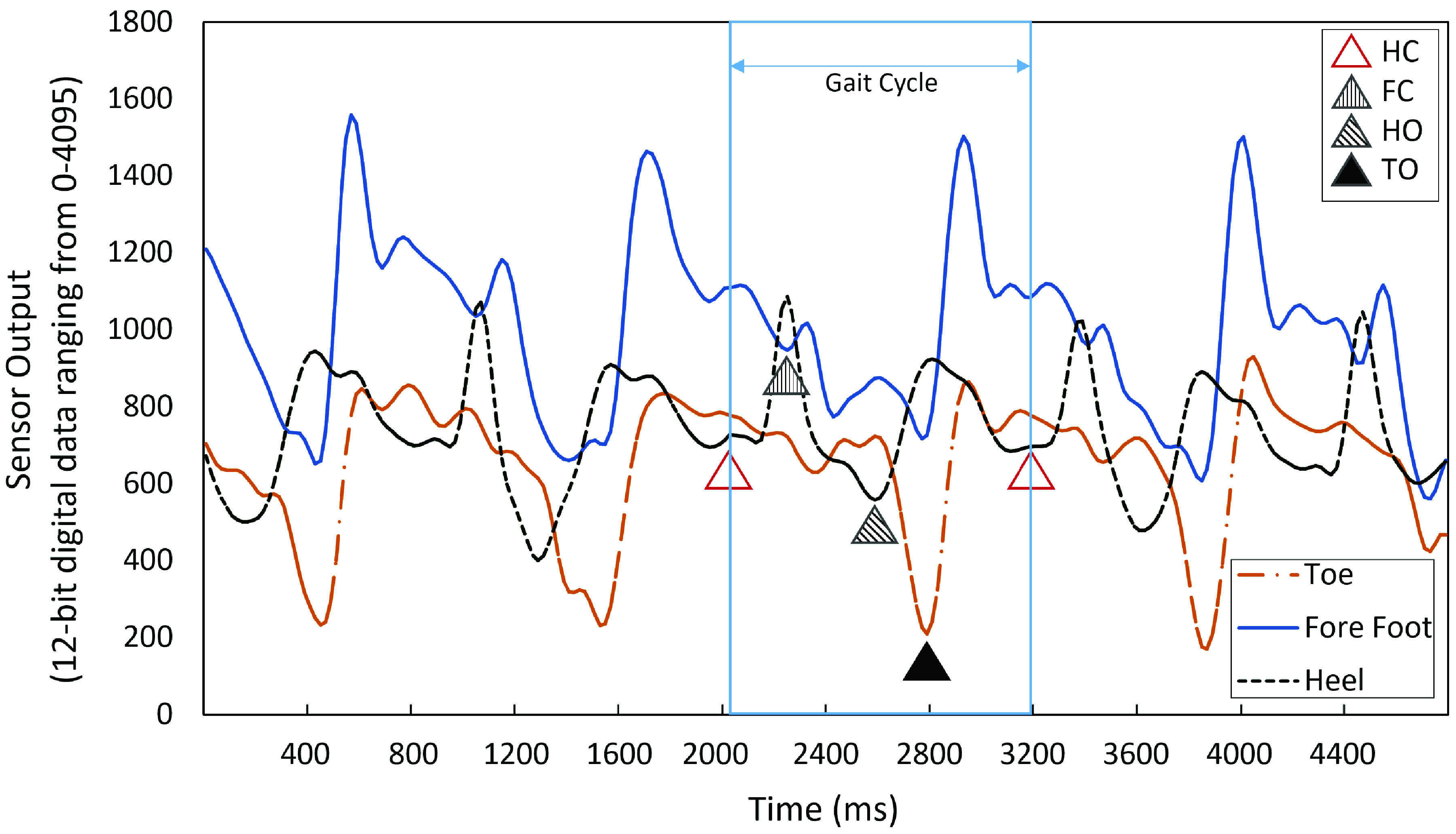
Illustration of the sensor outputs of the toe, fore foot, and heel sub-areas during gait. The identified key gait events based on the detection algorithm are marked by $\Delta $. HC-heel contact; FC-Forefoot Contact; HO-Heel Off; TO-Toe Off.

Fig. 7 shows exemplary sensor output trajectories of the defined sub-areas with the identified key events based on the detection algorithm.

### D. Experiments

Eight young male participants (age=24.0±1 years,

height =173.6±3.7 cm, weight=63.3±5.1 kg) were involved in an experiment. All the participants had the shoe size of US 9 (foot length=25.8±0.4 cm). They all self-reported without any disease that would affect their normal gait pattern. The experimental protocol was approved by the Institutional Review Board of Shenzhen University on January 17, 2019 (Approval #: 20190012). Informed consent was obtained from all participants.

**FIGURE 8.**
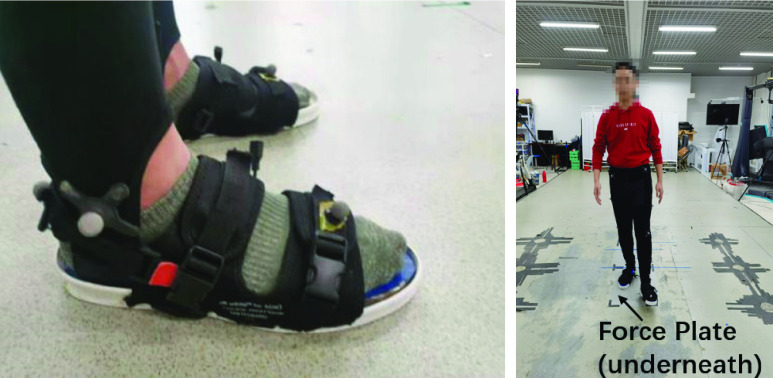
A participant wearing the instrumented shoe system and the overview of the experiment set-up.

Two force plates (Bertec, Sweden) placed underneath the walking platform with approximately 1 cm apart along the progression direction were used to obtain the ground reaction forces, which were in turn used as the reference measurements for the evaluation of the proposed gait phase detection algorithm. The force plates were synchronized with the instrument shoe at a sampling frequency of 50Hz. Prior to data collection, the participants were asked to wear the instrumented shoe system (see Fig. 8) and walk on a 10m-long walking platform with their comfortable walking speed for at least ten minutes. This allowed them to get familiar with the equipment and helped the experimenter to determine the starting point so that each participant could have a complete stance phase captured by a single force plate.

At the beginning of data collection, the participants were asked to step one foot on and off the force plate for three times. The resulted signals from the instrumented shoe system and the force plate were used for synchronizing these two systems. After that, the participants were asked to carry out straight-line walking and turning walking in different trials on the walking platform with their comfortable walking speed. In the straight-line walking trials, the participants started from the starting point and stopped at the end of the walking platform. The distance between the starting point and the force plate was approximately seven meters. A revious study [32] showed that such distance allowed the participants to reach a steady-state speed. Each participant repeated straight-line walking for five minutes. Ten gait cycles where the participants had a complete stance phase (right foot) on a single force plate were randomly selected for further analysis. In the turning walking trials, the turning routes were marked by color tapes on the ground which guided the participants to make a left 90° step turn (i.e., a change in direction opposite to the stance limb [33]) on the first force plate without stopping. Similar to the straight-line walking, each participant repeated turning walking for five minutes. Ten turning gait cycles were randomly selected for analysis. In total, data from 160 gait cycles were used for the system evaluation.

The raw data were transmitted wireless to a PC (Lenovo model Y50p, Intel Core i5 2.9GHz, 8GB RAM, with Intel Bluetooth module version LMP6.1280) via Bluetooth series communication, and the gait phase detection algorithm was run on the PC. The sampling data rate was 50 Hz, and the required data transmission speed was approximately 83200 bytes/s. Thus, the Baud rate was set at 921600 bps to ensure sufficient wireless data transferring speed. For sensor conditioning, the raw data obtained from each sensing unit were filtered using a third-order, zero-phase lag, low-pass Butterworth filter with the cut-off frequency of 7 Hz. The filtered data were recorded when no external load was applied on the instrumented shoe for approximately ten seconds. The mean data output from each sensing unit within this period of time was calculated. This was registered as the zero-off-set and later deducted from the actual sensor output [34].

### E. Evaluation

**FIGURE 9.**
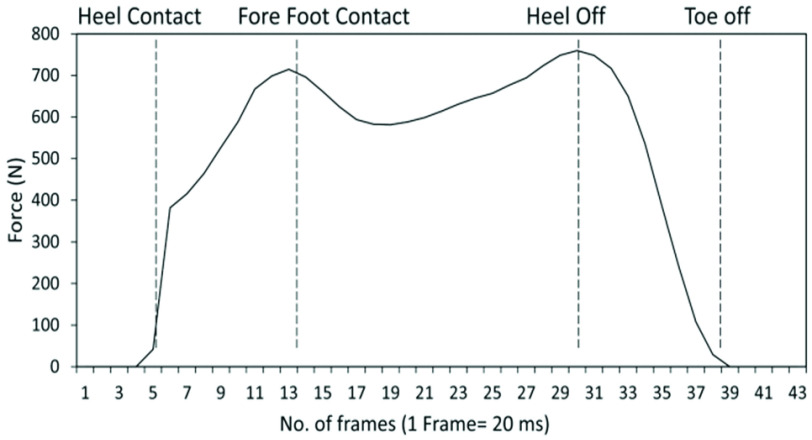
Key temporal gait events determined by the vertical ground reaction force.

The sensor output collected from the experiment was transferred and stored in the PC. The above-described gait phase detection algorithm was implemented based on these sensor output data with by a custom Python script (Python version 3.7.9). The references of the key temporal gait events were obtained according to the vertical component of the ground reaction force measured by the force plate, following the approach proposed in [35]. Specifically, the times of HC and TO were determined when the vertical ground reaction force increased above 10 N and decreased below 10 N, respectively., the time of FC was determined as the first peak in the ground reaction force, and the time of HO was determined as the second peak in the ground reaction force. Fig. 9 shows an exemplary trial of the vertical ground reaction force for the specification of key gait event references.

The relative difference (RD), mean absolute difference (AD), and the median error (ME) were calculated between the times of events detected by the instrumented shoe system $(t_{shoe})$ and the references ( $t_{ref}$). These parameters were calculated as follows.

\begin{align*} RD&=t_{shoe} -t_{ref} \tag{12}\\ AD&=\vert t_{shoe} -t_{ref} \vert \tag{13}\\ ME&=median(\vert t_{shoe} -t_{ref} \vert) \tag{14}\end{align*}

The RD represents the arithmetic error of the shoe-detected event timings compared to the references [36]. A positive value suggests a time lag of the detected event, and a negative value suggests an early detection (i.e., time lead) of the event. The AD, on the other hand, represents the magnitude of the error regardless of the direction [37]. The ME is the median value of absolute difference [16].

The durations of stance sub-phases were estimated by the determined gait events. The RD, AD and ME were calculated between the durations of each phase detected by the instrumented shoe system and by the references across the ten gait cycles. The percentage difference (%D) was used to quantify the errors of the estimated duration of each phase, which was calculated as the AD divided by the reference duration (*t_st_ref_*) [16].

\begin{equation*}{\%{ }}D=\frac {AD}{t\_{}st_{ref}}=\vert \frac {t_{shoe} -t_{ref}}{t\_{}st_{ref} }\vert \times 100\% \tag{15}\end{equation*}

The above calculations and data analysis were done by a custom Python script. Note that we did not evaluate the duration of the swing phase mainly due to two reasons. First, the swing phase is defined as the interval between TO and HC. In cyclic gait trials, knowing the performance of the proposed detection algorithm in detecting TO, and HC will help precisely evaluate the performance of the algorithm in detecting the swing phase. Second, the force plate can only measure one complete stance phase. Thus, we cannot determine two consecutive reference HCs, which makes the reference swing duration unavailable for evaluation.

## III. Results

The detection of the key gait events and gait sub-phases was done offline with the signals obtained by the instrumented shoe from the experiment and the gait phase detection algorithm. All the key gait events (i.e., heel contact, fore foot contact, heel off, and toe off) and gait sub-phases (i.e., initial contact, flat foot, and push off) under the testing trials were successfully detected, suggesting 100% detection rate.

The detection performance was further evaluated by the measurements obtained from the force plate which served as the benchmarking system. Given that the data output rate was 50Hz, the temporal accuracy level was 20ms. Table 1 shows the results of gait event detection. For straight-line walking, the ADs between the estimated time of each gait event and the reference measurement ranged from 1.9 to 2.8 data frames (i.e., 38–56 ms), and the MEs were within 2-2.5 data frames (i.e., 40–50 ms). The estimated times of HC and FF had a positive sign in the RDs, indicating that the detection was slightly delayed compared to the references. In contrast, early detections were generally found for the estimated times of HO and TO. Nonetheless, the magnitude of the RDs were all within 2 data frames (i.e., 40 ms).

**TABLE 1:** Evaluation results for gait event detection compared to the reference. (Gait events detected by the force plate as the ground truth.)

	Straight-line walking (Mean±std.)			Turning walking (Mean±std.)		
	AD frames (ms)	RD frames (ms)	ME frames (ms)	AD frames (ms)	RD frames (ms)	ME frames (ms)
HC	2.0±1.0	2.0±1.0	2.0	2.0±0.6	2.0±0.6	2.0
	(40±19)	(40±19)	(−40)	(39±11)	(39±11)	(−40)
FF	1.9±1.7	0.5±2.5	2.0	2.5±2.3	1.5±3.1	2.0
	(38±33)	(10±50)	(−40)	(50±45)	(30±61)	(−40)
HO	2.8±2	−1.6±3	2.0	3.6±2.5	−1.5±4.1	4.0
	(55±39)	(−31±60)	(−40)	(72±49)	(−30±82)	(−80)
TO	2.8±2	−0.9±3.3	2.5	4.2±2.7	−1.6±4.7	4.0
	(56±39)	(−17±66)	(−50)	(83±54)	(−31±94)	(−80)

AD=absolute difference, RD=relative difference, ME=median of the error, ms= mini-seconds.

The errors slightly increased during turning. The ADs ranged from 2.0 to 4.2 data frames (i.e., 40–83 ms) for turning walking, with the largest AD at TO (4.2±2.7 data frames). Similar to the straight-line walking, the estimated times of HC and FF had a positive sign in RDs, indicating that the detection was slightly delayed compared to the references. In contrast, early detections were generally found for the estimated times of HO and TO.

Table 2 shows the results of the estimated durations of the gait phases. For straight-line walking, the ADs ranged from 2.3 to 2.9 data frames (i.e., 45–58 ms). The results showed that, in general, the estimated durations of IC and FF were shorter than the references as indicated by the negative sign in RDs. In contrast, the estimated duration of PO was slightly longer than the reference. The MEs were about 2–3 data frames (40-60 ms). The percentage difference for each phase ranged from 5.48% to 5.88%. For turning walking, the ADs ranged from 2.6 to 3.9 data frames (i.e., 51–77 ms). The estimated durations were shorter than the references for all the phases of stance, as indicated by the negative sign in RDs. The MEs ranged from 2 to 3 data frames (40-60 ms), which was the same as the straight-line walking. The percentage difference of each phase ranged from 5.13% to 5.56%.

**TABLE 2:** Evaluation results for gait phase duration estimation compared to the reference. (Gait phase estimated from the force plate as the ground truth.)

	sub stance phase	AD frames (ms)	RD frames (ms)	ME frames (ms)	Error in percentage (%)
Straight-line walking	IC	2.3±1.6 (45±32)	−1.6±2.3 (−31±46)	2 (40)	5.88
	FF	2.9±1.8 (58±36)	−2.1±2.8 (−41±55)	3 (60)	8
	PO	2.3±1.8 (46±36)	0.8±2.8 (15±57)	2 (40)	5.48
Turning walking	IC	2.6±1.7 (51±34)	−0.5±3.1 (−9±61)	2 (40)	5.56
	FF	3.9±2.8 (77±55)	−3±3.7 (−59±74)	3 (60)	8.63
	PO	2.7±1.9 (53±38)	−0.1±3.3 (−1±65)	2 (40)	5.13

AD=absolute difference, RD=relative difference, ME=median of the error, ms= mini-seconds.

## IV. Discussion

This study presented a novel instrumented shoe system that can detect gait phases based on foot plantar pressure data. As being unobtrusive, our instrumented shoe system would not lead to discomfort for its wearers. There are a few low-cost pressure sensing insoles reported previously. For example, Shu et al. [48] proposed an in-shoe plantar pressure measurement system with 15 sensing units covering the corresponding anatomical areas. Zhao et al. [49] designed a flexible sensor matrix film that involved 16 piezoresistive sensing units. Martini et al. [16] designed a pressure-sensitive insoles with 64 sensing units. Plus, Chen et al. [50] designed a piezo-resistive fabric material based insole which consists of 96 pressure sensors. Compared to these previous systems, there are 174 sensing units with our proposed instrumented shoe, which provide much higher sensing resolution compared to the existing low-cost instrumented shoe systems. Higher sensing resolution suggests higher precision of the plantar pressure data. Therefore, we argue that our proposed system can serve as a better solution to low-cost plantar pressure measurement and assessment than the existing ones.

In gait phase detection applications, what is more important is knowing when the gait phase starts, when it ends and how long it lasts, rather than just knowing whether it occurs. Gait sub-phases are determined by key gait events as specified in the present study, and their durations are the time intervals between the key gait events defining them. Thus, we used detection time error measures (i.e., AD, RD, ME, and %D) reflecting the gait event and phase detection time differences between our proposed system and the reference measurement system (i.e., force plates) in performance evaluation. The results showed that the mean absolute detection time errors ranged from 38 to 58 ms during straight-line walking. Similar ranges of detection time errors have been reported in earlier studies. For example, Martini et al. [16] reported that their pressure sensitive insoles had mean detection time errors ranging from 40–60 ms for the key gait events, which could lead to similar levels of errors in the sub-phase duration estimation. The eSHOEs system developed earlier [12] had detection time errors ranging from 29–46 ms. Smaller errors in the gait events/phases detection have been reported earlier, which could be as low as milliseconds [21], [22]. However, most of them focused on detecting heel strike and toe off, or the stance and swing phases only, while our proposed system can be used to detect sub-gait phases. For commercial systems, previous studies demonstrated that the Medilogic Ⓡ insoles can estimate the stance phases with the detection time error at approximately 10% of stance duration [40]. Another study showed that the F-scan Ⓡ insoles had a 20–30 ms delayed detection compared to the data of a force plate [41].

By comparing the performance of our proposed system with the abovementioned published results, we could tell that the detection time errors of our system were within an acceptable range. Nevertheless, it is worth noting that there existed differences in the experimental design, evaluation parameters, and targeting gait events/phases between our study and other studies. Thus, to draw more convincing conclusions, comparisons should be done between studies with consistent evaluation protocols. This could be one possible direction for future research.

This study offered a practical solution to eliminating the cross-talk effect in the networked sensing-array structure. The matrix design of the pressure sensing arrays makes the instrumented system compact and easily embedded with the shoe sole. However, the cross-talk effect inevitably exists in such structure. This study showed that such cross-talk effect can be eliminated by the zero penitential method. Since the zero penitential method can be scalable to different numbers and types of sensing arrays (e.g., piezoelectric sensors), this practical method can shed a light for future relevant studies.

Unlike extant force-sensor-based insole/shoe systems where gait phase detection was mainly based on the output from isolated sensor(s), we developed a novel gait phase detection algorithm based on foot plantar pressure data. More specifically, this algorithm depends on multiple sensing arrays collectively in different sub-areas of the foot plantar surface. In the previous studies, key gait events were usually detected by one single sensing unit or a couple of discretely distributed sensing units [5], [6], so the detection accuracy was highly dependent on the locations of the sensing units. In contrast, using foot plantar pressure data can provide a greater insight into feet-floor interaction, which can help reveal gait patterns more accurately than the isolated sensing units.

The novel gait phase detection algorithm was designed to search the peak heuristic of the pressure sensing data. Similar peak heuristic approaches have been proposed in some earlier gait phase detection studies [42]. However, previous studies were mainly based on IMU signals. The results in the present study showed that this peak heuristic search algorithm worked well for pressure sensing data. One advantage of the peak heuristic search algorithm is its high computational efficiency. Compared to the machine learning methods, the peak heuristic search algorithm did not require a large set of training samples. Instead, it was developed based on the relevant domain knowledge. It can be seen from the pseudo code (in the supplementary document) that this rule-based algorithm was straightforward and can be easily distributed on the onboard system. Thus, it is feasible to apply this algorithm in real-time gait phase detection.

Besides straight-line walking, this algorithm was also evaluated for turning gait. Turning gait can bring additional challenges when developing IMU-based gait phase detection models, because the change of walking direction can result in more intensive IMU signal changes compared to straight-line walking [43]. However, this would not be a problem for force-sensing-based gait phase detection methods. Our results in deed showed that the proposed algorithm worked well for turning gait. The mean detection errors in gait phase estimation during turning walking were between 51 and 77 ms. Like the straight-line walking, the errors during turning gait were also within the acceptable range, which showed that this instrumented shoe system can be more versatile than previously reported IMU-based systems.

This instrumented shoe system was developed based on off-the-shelf low-cost FSF known as the Velostat, which is a conductive polymer composite consisting of carbon- impregnated polyethylene [44]. Apart from its low cost, this FSF has demonstrated its unique advantage. Specifically, this thin film pressure sensor was softer and thinner than the FSR or optoelectronic pressure sensors. Thus, they can be less intrusive than the other types of pressure sensors and be easily integrated into the shoe soles.

When fabricating the sensing arrays, the force-sensitive film was sandwiched by two layers of conductive tapes. This structure can be viewed as a mini capacitor. The electric field between the closed sensing units might cause electric charges stored on them which might induce the capacitive effect. However, given that the permeability (dielectric constant) of this kind of carbo-impregnated polyethylene composite was approximately 3–6 (depending on the volume fraction of the carbon fiber) [45], the capacitance of each sensing unit can be estimated to be approximately 0.06-0.13 nF. This suggests that the capacitive effect can be neglected. Additionally, the frequency of input signals was typically less than 1Hz (in accordance with the stepping frequency during gait), which was considered low. Given this, the delay in the output signal resulting from the parasitic capacitance can be negligible. This was also evidenced in the sensor characteristic test with cyclically applied forces (Fig. 5), where no delay in the sensor output can be observed with respect to the changing force.

By providing important temporal gait information, this instrumented shoe system can be used in several clinical and scientific applications. For example, it can be used in pathological gait diagnosis, gait rehabilitation evaluation, and control of lower-limb robotic assistive devices. Pappas et al. [46] proposed an automatic gait assistive system which detected gait phases by both the force sensors and gyroscope, and used such information to control electrical stimulation in order to assist people with injured spinal cord. Early studies also suggested that the detection latencies of up to 150 ms were acceptable for online functional electrical stimulation [22], [23]. The detection errors of our algorithm were about 38–83 ms, which were within the acceptable range.

Several factors may have contributed to the detection errors. First, as indicated in the characteristic test, due to the inherent limitations of the sensing array, the relationship between the sensor output and input cannot be perfectly linear. Though we adopted collective output from multiple sensing units to reduce the effects of nonlinearity, such effects cannot be completely avoided. Second, there is manually-induced unevenness of the FSF-tape-insole interface, which would bring noise to signals for gait phase detection. Thus, to improve detection accuracy, standard manufacturing processes should be adopted to replace manual operations in assembling the sensing structure. Third, detection errors may be affected by the data frame intervals. Although the current data output rate (i.e., 50 Hz) can meet the minimal requirement for capturing the plantar pressure dynamics associated with typical walking patterns [47], it could be further increased to shorten data frame intervals and reduce detection errors. A possible way to increase data output rate is to process the raw data onboard and only send out key gait event data wirelessly.

There existed some other limitations in the present study. One is that the swing phase duration was not reported in this study, despite that the swing phase duration can be estimated as the time difference between toe off and subsequent heel contact. The reason for this limitation is that the force plate system we used only allowed us to register one complete stance phase, which made it impossible to have reference measurement for the subsequent heel contact. To address this problem, future research should be conducted with a modified force plate system that allows for multiple stance phase registrations. Another limitation is that only one single size (US 9) of the instrumented shoe was tested. Other shoe sizes will be tested in the future to better validate the proposed gait phase detection system. In addition, instrumented shoe system was only tested on healthy participants. To be more clinically significant, patients with pathological gait should be included when evaluating the gait event detection performance of this system in future research. Lastly, though the increased number of sensing units can lead to higher resolution in measurement, it may also require higher power consumption which may limit the practical applications of the proposed system. Thus, there is a need to figure out the relationship between power consumption and the number of sensing units in future research.

## V. Conclusion

This study presents an instrumented shoe system that can detect gait phases by foot plantar pressure data. Experimental results showed that the accuracy of the proposed gait phase detection algorithm is within the acceptable range for both straight-line walking and turning walking. This low-cost and portable instrumented shoe system has high wearability, making it suitable for personal use. It has significant potentials for translational engineering applications in healthcare and telemedicine. By allowing convenient gait monitoring outside of clinical settings, it can help translate gait-related research from lab to home healthcare settings. This kind of wearable technology will enable more extensive data collection on patients during activities of daily living. The data can provide deeper insights into movement disorders and facilitate developing more effective treatment and rehabilitation strategies. The instrumented shoe system also has the potential to aid in developing personalized care solutions and monitoring treatment outcomes over time. Overall, it represents an innovative wearable platform that can help accelerate the translation of bioengineering and medical research insights into practical tools and approaches to improve patient care and outcomes.
